# A New Test Paper, Suggested by Private J. C. Wharton, of Atlanta, Georgia

**Published:** 1864-04

**Authors:** 


ART. II—*A New Test Paper, suggested by Private J. C. Wharton, Atlanta, Ga., detailed in Purveying Department.*

In the course of a number of experiments with the extract of logwood, some singular properties were observed, of which the following may prove of interest and value:

It was found that by adding to a solution of extract of logwood (consisting of one ounce to one pint of water) an equal volume of ox-gall, exposing the mixture for several hours to the rays of the sun, and then setting it aside for three or four days, a compound was produced very sensitive to the action of both acids and alkalies—the former making a crimson or yellow change, as the acid is strong or weak, while the latter renders the red color of the solution violet or blue. This property would seem to make it valuable as a test for both, as it is only necessary to prepare paper with it in the usual manner, by spreading the compound upon it. It possesses the combined virtues of both litmus and turmeric, and is apparently as sensitive as either.

What change the bile produces in the hæmatin (which is beyond doubt the active or sensitive principle) it is hard to determine, unless it be that the small amount of free alkali in bile serves to give the blood-red color to the compound, which is thereby rendered a better ground for the development of the colors (red, yellow or violet) which acid or alkali produces. There is an objection which might be urged against it as an infallible test. It is that, on account of the tannic acid in the extract, some metallic salts, as of iron, copper, &c., re-act in a manner somewhat similar in appearance to alkalies. This may be guarded against by adding to the preparation a few grains of ferrocyanide of potassium (which must be freed from alkalinity), when it will become an easy matter to distinguish them, as ferrocyanides of the metals will be formed on the paper, while alkalies are unaffected by it. Some striking results take place when solutions of several of the salts are applied, and may be worthy of note. Borax re-acts at first as a decided alkali, and soon afterwards the paper is bleached, a margin of violet being left around a white spot. This effect is so remarkable as to characterize it from all other salts yet tried, and may prove to be a sure test of borax and boracic acid. Oxalic acid also acts in a peculiar way, showing an acid re-action with a dark margin. The marginal lines in many other instances are of importance, indicating frequently a combination of two substances (as in sesquichloride of iron) and sometimes of three (when an acid and two or more metals are combined).

Another singular fact may as well be mentioned: The odor of musk or amber is quite evident in the solution, and shows a chemical change which is worthy of investigation.

P. S.—Since the report concerning test paper was made, further facts have been observed which are seemingly so uniform in their occurrence as to merit a careful examination. The principle of these are the changes produced by the action of the three mineral acids, viz: sulphuric, nitric and hydrochloric, as also by different admixtures of them, all of which, when strong, form spots of such characters as to distinguish each from the other. This may be seen by letting fall upon the paper a drop of each, and need not be described. Nitric acid re-acts in a manner which, considering the difficulty that has always existed in testing for it and its salts, is most important and valuable if it proves peculiar to it. Even a few drops in a thousand or more of sulphuric acid make the distinguishing mark, known by its greenish yellow centre with a crimson margin. The paper is rendered more sensitive to this change by first moistening it with the tongue. In testing for nitrates it is best to wet the paper with a solution of the salt (as, for instance, nitrate of potassa), and then apply a drop of sulphuric acid, when its central spot and marginal lines will indicate the presence of nitric acid which is set free. If chlorate of potassa be used instead of the nitrate, the appearance will be so nearly the same as to be mistaken for it.

This is a point upon which more light is needed, and, it is hoped, will engage the attention of Southern chemists.

In using the paper the changes, which in some cases are not immediate, will be hastened by a gentle heat. Its hygrometric moisture may also be driven off by gentle heat in others to advantage, and sometimes increased by the saliva or a little water.

[The logwood, a vegetable red, renders the paper sensitive to alkalies; the acid re-actions takes place with the various elements of bile, and these will be so numerous as to produce confusion and diminish the value of the test paper. The odor of musk is entirely due to the bile and is always noticed in the preparation of inspissated ox gall.—ED]

